# Protective Effect of Melatonin Administration against SARS-CoV-2 Infection: A Systematic Review

**DOI:** 10.3390/cimb44010003

**Published:** 2021-12-22

**Authors:** Antonio Molina-Carballo, Rafael Palacios-López, Antonio Jerez-Calero, María Carmen Augustín-Morales, Ahmed Agil, Antonio Muñoz-Hoyos, Antonio Muñoz-Gallego

**Affiliations:** 1 Department of Pediatrics, Medicine Faculty, University of Granada, 18071 Granada, Spain; amolinac@ugr.es (A.M.-C.); aejerezc@gmail.com (A.J.-C.); amunozh@ugr.es (A.M.-H.); 2 “Clínico San Cecilio” University Hospital, 18071 Granada, Spain; 3 Biosanitary Research Institute of Granada (ibs.Granada), University of Granada, 18071 Granada, Spain; aagil@ugr.es; 4 Health Center “Las Gabias”, Granada Metropolitan District, 18071 Granada, Spain; centellito@correo.ugr.es (R.P.-L.); mcaugustin6@yahoo.es (M.C.A.-M.); 5 Department of Pharmacology and Neurosciences Institute, School of Medicine, University of Granada, 18071 Granada, Spain; 6 Languages and Computer Science Department, University of Malaga, 29003 Malaga, Spain

**Keywords:** COVID-19, melatonin, SARS-CoV-2, prevention

## Abstract

Introduction: according to the World Health Organization (WHO), COVID-19 is an infectious disease caused by the SARS-CoV-2 virus, responsible for an increasing number of cases and deaths. From a preventive and therapeutic point of view, there are two concerns that affect institutions and healthcare professionals: global immunization (which is still far from being achieved) and the availability of drugs capable of preventing its consequences in the infected patient. In this sense, the role that melatonin can play is has been assessed in the recent literature. **Justification and Objectives**: the serious health, social and economic consequences of COVID-19 have forced an urgent search for preventive methods, such as vaccines, among others, and therapeutic methods that could be alternatives to the drugs currently used. In this sense, it must be accepted that one of the most recommended has been the administration of melatonin. The present study proposes to carry out a systematic review of its possible role in the treatment and/or prevention of COVID-19. **Material and methods**: a systematic review of the literature related to the prevention of COVID-19 through the administration of melatonin was carried out, following the sequence proposed by the Prisma Declaration regarding the identification and selection of documents, using the specialized health databases Trip Medical Database, Cochrane Library, PubMed, Medline Plus, BVS, Cuiden and generic databases such as Dialnet, Web of Science and Google Scholar for their retrieval. Appropriate inclusion and exclusion criteria are described for the articles assessed. The main limitation of the study has been the scarcity of works and the lack of defining a specific protocol in terms of dosage and administration schedule. **Results**: once the selection process was completed, and after an in-depth critical analysis, 197 papers were selected, and 40 of them were finally used. The most relevant results were: (1) melatonin prevents SARS-CoV-2 infection, (2) although much remains to be clarified, at high doses, it seems to have a coadjuvant therapeutic effect in the treatment of SARS-CoV-2 infection and (3) melatonin is effective against SARS-CoV-2 infection. **Discussion**: until group immunization is achieved in the population, it seems clear that we must continue to treat patients with SARS-CoV-2 infection, and, in the absence of a specific and effective antiviral therapy, it is advisable to continue researching and providing drugs that demonstrate validity based on the scientific evidence. In this regard, we believe that the available studies recommend the administration of melatonin for its anti-inflammatory, antioxidant, immunomodulatory, sleep-inducing, CD147, Mpro, p65 and MMP9 protein suppressing, nephrotoxicity-reducing and highly effective and safe effects. **Conclusions**: (1) melatonin has anti-inflammatory, antioxidant, immunomodulatory, and Mpro and MMP9 protein-inhibitory activity. (2) It has been shown to have a wide margin of safety. (3) The contributions reviewed make it an effective therapeutic alternative in the treatment of SARS-CoV-2 infection. (4) Further clinical trials are recommended to clearly define the administration protocol.

## 1. Introduction

The COVID-19 coronavirus pandemic, responsible for the “severe acute respiratory syndrome” SARS-CoV-2, continues to rise around the world. As of today, 11 August 2021, a total of 250,082,536 cases have been collected, with 5,052,991 deaths, despite 7,277,064,480 doses of vaccines having been administered [1,2,3,4].

Approximately 40% to 45% of people infected with this coronavirus remain asymptomatic [5,6], and most of them (about 80%) can recover from the disease without treatment [7]. Therefore, the actual number of COVID-19-infected cases is assumed to be much higher than what has been reported [8,9]. However, the level of antibody seropositivity in the general population is still low [10,11], indicating that most of the world’s population remains susceptible.

Most patients have shown an adequate antibody response after infection [10], and proven reinfection has been rare [12]. Moreover, transfusion of convalescent plasma has been indicated as an effective therapy against COVID-19 infection [13]. This evidence, together with other evidence of various characteristics, recommended initiating the development of vaccines against COVID-19. The genome of this coronavirus contains four main structural proteins: the spike protein (S), membrane (M), envelope (E) and nucleocapsid (N). The main target of the antigenic epitopes of the COVID-19 vaccine are the S protein [14]; the S1 domain, which contains the receptor-binding domain (RBD) for the host cell receptor angiotensin-converting enzyme-2 (ACE2) [15]; the N-terminal domain (NTD), which has been demonstrated as another site with potent neutralizing activity [16,17,18]; the S2 domain, which contains the fusion peptide [19,20].

A total of 63 different vaccines have been registered in clinical studies at different stages, and 170 in pre-clinical studies. Of these, Pfizer-BioNTech’s vaccine is 95% effective, Moderna’s is 94% effective and AstraZeneca-Oxford’s is 59% effective, showing an adequate safety profile [21], without forgetting the reality of some adverse effects, such as thromboembolic phenomena, among others [22,23,24,25].

Nonetheless, vaccines to prevent COVID-19 infection are considered the most promising approach to curb the pandemic. In this regard, different strategies are being studied, including blocking SARS-CoV-2 from binding to human cell receptors, preventing the synthesis and replication of viral ribonucleic acid (RNA), restoring host innate immunity, modulating specific immunity, and suppressing excessive inflammatory responses [26,27,28,29,30,31].

Despite these achievements, the current circumstances arising from the irregular distribution of vaccines, transport and storage problems and the difficulties generated by the so-called “anti-vaccine” groups, mean that we are still far from achieving the desired global herd immunity.

**Figure 1:**
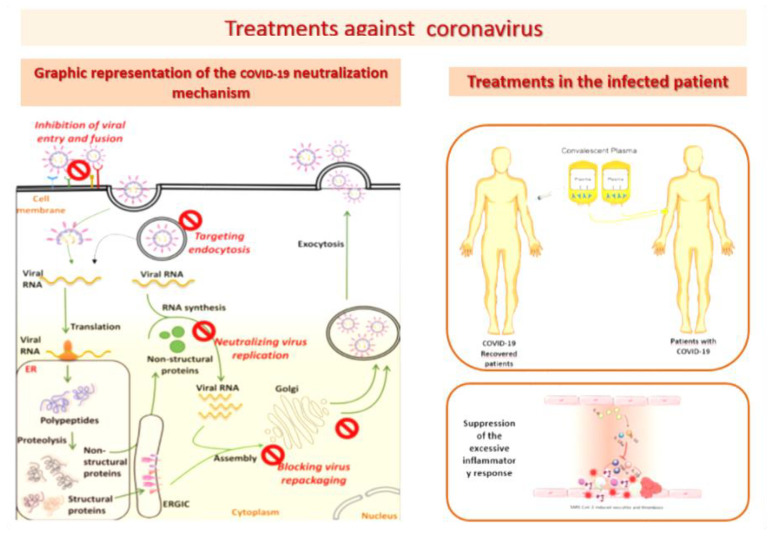
Treatment Against SARS-CoC-2.

**Figure 2:**
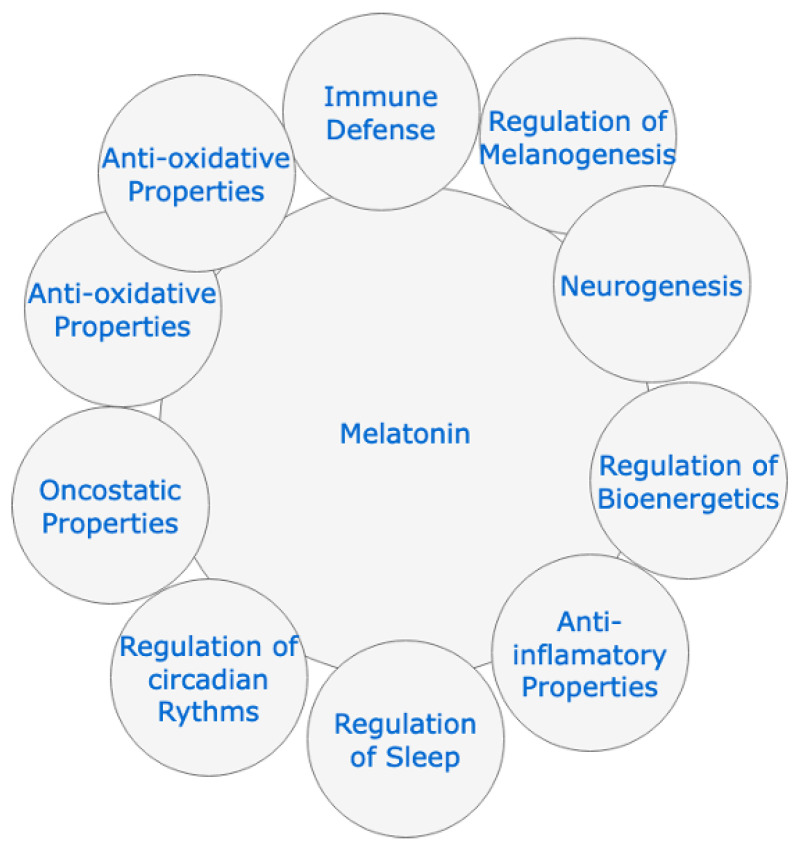
Physiological effects of melatonin.

This invites further research to find treatments that are effective, with few side effects and affordability for different economies, as Figure 1 depicts. While these difficulties are being resolved, it is necessary to reduce the morbidity and mortality of this pandemic by using alternative treatments such as melatonin [32]. Among its functions, as Figure 2 depicts, it modulates cell function by activating intracellular signaling pathways and the transcription factors involved in them, and is capable of dampening inflammatory activity, acting as a potent antioxidant, optimizing the functions of the mitochondria, boosting immunity, has the capacity to reduce circulating cytokine levels and inhibits the CD147 signaling pathway, thus preventing SARS-CoV-2 from entering cells [33,34,35,36]. In addition, melatonin is a hormone that is safe to administer even at high doses, with protective effects through peroxyl radical scavenging and glutathione activation [37,38,39,40,41]. 

Doses above 500 mg of melatonin have been shown to be useful in dampening the initial cytokine storm caused by SARS-CoV-2, and this dose is well tolerated in humans. Previously, its usefulness in mitigating the symptomatology and lethality associated with other viral infections, including influenzaviruses and SARS-CoV, and its prophylactic role, particularly in patients with preconditions of suppressed melatonin synthesis, have been noted [32].

Different authors [33,35,36,38,39,41,42,43,44,45] recommend the administration of melatonin for its anti-inflammatory, antioxidant and immunomodulatory capacity, mechanisms that could counteract or dampen the production of the responsible cytokines of the “inflammatory storm” and of the major complications that have been described in the disease caused by SARS-CoV-2.

Ultimately, the objective of this work has been to carry out a review of the medical literature and to analyze the knowledge and evidence that exists after the use of melatonin in the treatment of SARS-CoV-2 infection.

## 2. Material and Methods

A systematic search process has been carried out in which the articles published by Urrútia and Bonfill [46] and Pardal [47] have been considered. This search was carried out using the following search engines:

- Trip Medical Database.
- Cochrane Library.
- PubMed.
- MedlinePlus.
- BVS.
- Cinahl.
- Cuiden.
- Dialnet.
- Scholar Google.

Before entering keywords in search engines, the question of P.I.C.O. was considered:

- Patient: people of any age and sex infected by the SARS-CoV 2 virus.
- Intervention: intervention program through the administration of melatonin to prevent infection by SARS-CoV 2 and the development of COVID-19.
- Comparation: compare the currently available treatment protocols of etiological active ingredients and vaccines with the administration of melatonin as a preventive of COVID-19.
- Outcomes: raise the results that are to be obtained through the proposed intervention to plan the objectives of the work, such as reducing the incidence of COVID-19 infection and the variability of the results according to parameters by population groups (age, sex and previous pathologies).

The keywords that have been entered in the search engines are: COVID-19, melatonin, SARS-CoV 2 and prevention, using the Mesh and DeCS corresponding to the identified keywords, as well as the Boolean AND/OR connectors. The filters and descriptors used in each database changed according to the format used in each one of them.

To narrow down the search, the following criteria have been applied:

Inclusion criteria:

(1) Full text documents.
(2) Language: Spanish and English.
(3) Less than 10 years old, except for those documents that present relevant information and that are not obsolete.
(4) Works focused on the administration of substances with preventive power of the COVID-19 infection, especially those that refer to melatonin.
(5) Case or group studies, books, articles published in prestigious scientific journals, conference proceedings and communications from official bodies in the health field.

Exclusion criteria:

(1) Documents without scientific validity.
(2) Documents with high risk of bias.
(3) Opinion articles.
(4) Studies with a follow-up time of less than 1 month.

The main limitation found to carrying out this work has been the scarce specific documentation in the databases explored, with a small number of articles focused on the protocols related to the intervention through the administration of melatonin in SARS-CoV-2 infection.

Once the systematic search was carried out and subjected to a rigorous critical reading, those documents that were repetitive or did not meet the indicated inclusion criteria were rejected, and 197 references were selected for their relevance, of which 40 papers were finally used.

**Figure 3:**
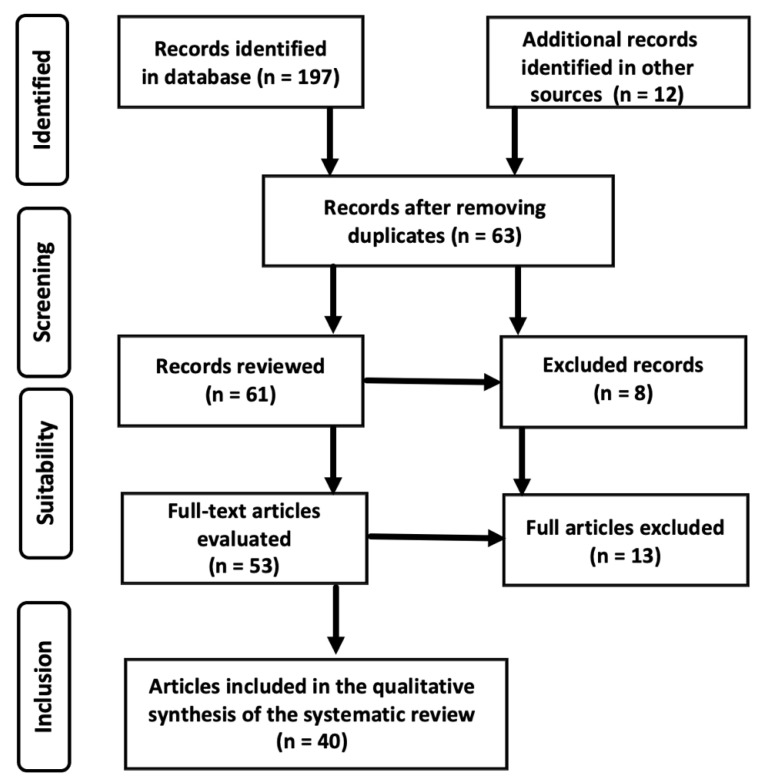
Criteria for article selection.

A flow chart with the criteria applied for the article selection process is shown in Figure 3. A total of 197 articles were identified in the initial search, in addition to another 12 articles from other sources. After eliminating duplicates and those that did not meet the inclusion criteria, we were left, in the selection process, with 63 articles. Of these, only 61 were reviewed, subsequently excluding another 8 articles, thus reducing the sample of papers to 53 full-text articles. Finally, due to the existence of other factors, another 13 papers that did not refer exactly to the subject matter of the review had to be excluded. Forty articles remained for the final analysis for the qualitative synthesis of the systematic review.

## 3. Results

Once the document selection process was completed, in Table 1 a summary of Databases reviewed is included, they were subjected to a careful critical analysis, obtaining the results that we present in Table 2. In each of its sections, the essential elements of the article under study are detailed, obtaining the following aspects as an analysis procedure: (1) authors of the work and journal that published the article; (2) objective that arises in the project; (3) the type of study; (4) quality of the same and level of evidence that it contributes *Q (A-Average/M-Medium/L-Low)*; (5) most relevant results; (6) conclusions that were obtained.

## 4. Discussion

The achievement of vaccines and their gradual administration to the population is transforming the global panorama of this pandemic; however, it is more than likely that, to achieve group immunity at the international level, a period will have to pass. Meanwhile, we will have to continue looking for treatments that help mitigate the damage that is occurring in non-immunized and especially sensitive patients (the elderly, immunosuppressed, sufferers of heart disease, cancer patients, diabetics, the obese, etc.).

As is known in the different protocols that have been applied in the Intensive Care Units to patients infected by SARS-CoV-2 with serious complications, among other therapeutic resources, antivirals and antibiotics from the group of macrolides have been included, as well as corticosteroids, biological therapies and even nonspecific intravenous immunoglobulins in severe cases (without clear evidence of their usefulness at doses of 1g/kg/day for 2 days or 400 mg/kg/day for 5 days), supporting that supportive treatments for basic vitality (ventilatory, cardiocirculatory, homeostatic and renal), together with specific measures (corticosteroids, tocilizumab, etc.), have demonstrated their usefulness and efficacy in numerous critical patients [52,53,54,55,56,57].

Thus, due to the lack of specific and effective active ingredients, there are numerous studies and recommendations that indicate the need for alternative treatments that cover the care and preventive needs of SAR-CoV-2 infection, such as those indicated by Anderson and Reiter [33], who recommend the administration of melatonin for its anti-inflammatory and antioxidant capacity, which leverages to buffer the cytokine storm, reducing the symptoms and lethality of the infection. It is also shown to be safe at high doses and to have a prophylactic effect.

These affirmations are corroborated by the works carried out by Karamitri and Jockers [35], which confirm the antioxidant capacity of melatonin through eliminating peroxyl radicals and activating glutathione, and by Alghamdi [36], which corroborates the anti-inflammatory, immunity-enhancing and reducing activity of the concentration of circulating and inhibitory cytokines of the CD147 pathway. Furthermore, Zhang et al. [41] state that it is highly effective in the elderly and immunosuppressed with problems synthesizing melatonin, undergoing chemotherapy or transplants, or undergoing chronic treatment with corticosteroids.

Along with these contributions that we consider important, well designed, and published in prestigious journals, we fail to understand how it has not been implemented in the clinic in a generalized way so that its usefulness, recommended dose and treatment guidelines can be assessed and defined, such as administration based on the circadian rhythm, etc.

Along with these initial works, the documents that have finally been selected to support this review have been those that are collected in Table 2, from which the following contributions, evidence and comments have been extracted.

Brusco L et al. [42] describe that deep sedation performed in the ICU in patients affected by SARS-CoV-2 increases long-term mortality. This indicates that the administration of melatonin reduces the use of sedation, pain, agitation, and anxiety, while it improves the quality of sleep. Furthermore, as a cytoprotective agent, melatonin restores the optimal circadian pattern of the sleep/wake cycle, which improves the clinical condition of the person with SARS-CoV-2 pneumonia. It is concluded in this work that the use of melatonin as adjunctive therapy in ICU patients is recommended, a statement that is supported by the reduction in the length of stay in the ICU. Added to this is its high margin of safety, the possibility of administration by different route—including oral, its stability without refrigeration and its low cost, which makes it accessible to geographic areas that lack specific infrastructures or to countries with limited resources. These are undoubtedly interesting considerations because it is a review of clinical trials, with a medium level of evidence. The mentioned study’s interest is mainly focused on the effects on sleep and a certain cytoprotective capacity, and a dose of 9 mg is proposed, clearly insufficient to achieve other effects in the evolutionary stages of the disease.

**Figure 4:**
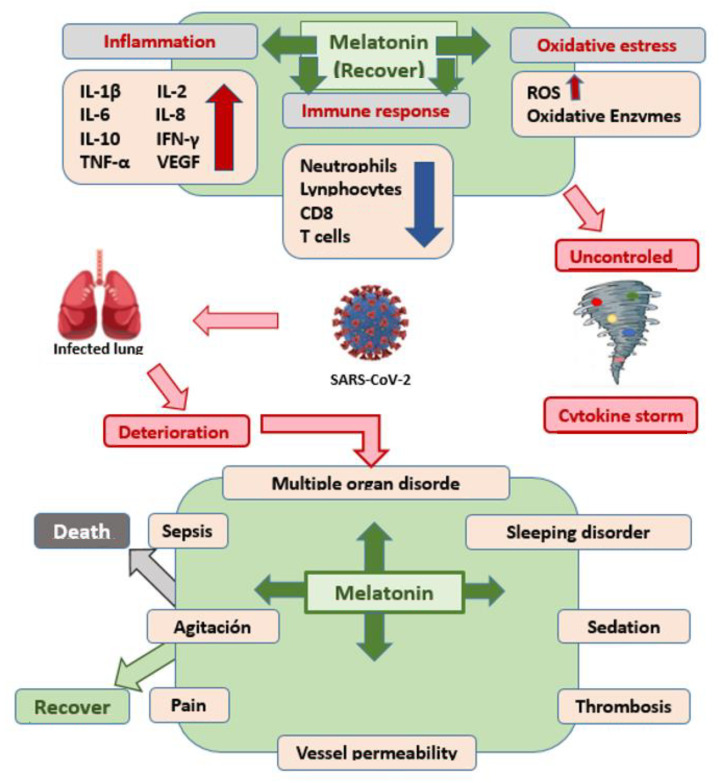
Mechanism of action of melatonin against SARS-CoV-2.

On the other hand, Cardinali D et al. [43] focus on the action of melatonin as a powerful chronobiotic with a slight hypnotic capacity, capable of synchronizing circadian rhythms, verifying that the prescription of melatonin two weeks before vaccination against SARS-CoV-2 improves the quality of sleep and guarantees that the vaccination is carried out in optimal conditions, keeping the patient’s mood and response capacity elevated and seroprotection. Added to this is its anxiolytic and antidepressant power, ultimately contributing to the effectiveness of the treatment. These are undoubtedly aspects of great interest, centered on its ability to regulate biological rhythms and, especially, the rhythm of sleep, which is altered in affected patients. In addition, it seems that its immunomodulatory and immune system enhancer role is being demonstrated again, an aspect that had already been previously referred to in the literature, precisely in seroconversion with a clear increase in the “booster” effect [53,54,55,56], as Figure 4 depicts.

In another interesting contribution [42], the beneficial effects of melatonin as an adjuvant in the treatment of SARS-CoV-2 infection are emphasized, which are related to its anti-inflammatory, antioxidant, and regulatory capacity of the immune response, as it is documented in respiratory disorders, shown in Figure 4.

**Figure 5:**
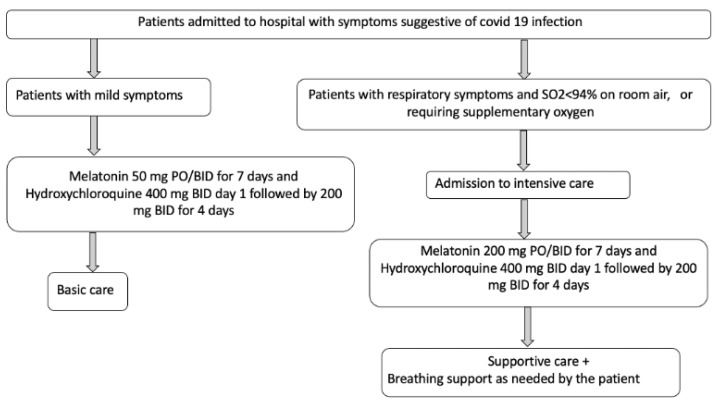
Melatonin administration algorithm against COVID-19. Inspired in Reiter RJ et al. [57].

Reiter RJ et al. [57] once again describe that high mortality is caused by the uncontrolled innate immune response and the intense inflammatory response, and melatonin can be administered as a prophylactic by regulating the innate immune response’s exaggerated and excessive inflammation, promoting adaptive immune activity. These investigators propose the following therapeutic algorithm shown in Figure 5 for its use in patients affected by the pandemic, suggesting administering a dose of 100–400 mg/day as a supplement, especially if effective antiviral treatment is not available:

This seems to be a very appropriate and promising proposal, with pathophysiological grounds. Perhaps the most novel are the regimen and the dose recommended by the authors. It has been published that, for melatonin to enter the mitochondria, minimum doses of 50 mg are necessary, and, in this case, it is more than enough.

There are studies with less specific proposals [56] which are limited to continuing life support treatment together with the joint administration of melatonin and tocilizumab. Zúñiga-Blaco BL et al. propose new guidelines for outpatient treatment in the early stages of infection [58].

The protective effect against encephalocarditis virus in mice had already been demonstrated and published years ago and, in this sense, more recently, other studies [59] have evaluated the antiviral activity of melatonin in mice inoculated with Semliki Forest virus and in mice stressed with attenuated non-invasive West Nile virus, 3 days before and 10 days after virus inoculation. A reduction in viremia and a significant delay in the onset of the disease and death between 7–10 days was observed, going from 100% to 44%, even decreasing to 20% if the dose was higher. The antiviral protective efficacy in mice has been demonstrated with the administration of melatonin, even with a certain dose dependence, an essential issue in humans, in which there is still a long way to go.

In the same sense, we have compiled other contributions [44] which describe the beneficial properties of melatonin administration in different viral infections, including viral respiratory disorders associated with oxidative stress, inflammation, and immune dysfunction. The management of oxidative stress and inflammatory responses, as well as the regulation of immune responses, may be decisive in the affectation of the respiratory tract caused by SARS-CoV-2. These authors propose that the use of melatonin be promoted due to its efficacy, wide margin of safety, low cost, and easy availability.

All the above leads to the assertion that melatonin is an active ingredient that alleviates the clinical symptoms of COVID-19, although it does not stop viral replication or transcription [37]. One of the factors that make older people more susceptible to suffering from the symptoms of this disease is the reduction in the endogenous production of this molecule, together with the increase in CD147 receptors. These authors propose the administration of melatonin in combination with antivirals since they produce a suppression of CD147, reduce the negative effects of antivirals and modulate the immune response.

The work developed by Feitosa et al. [38] highlights the important role of melatonin in the treatment of COVID-19 by acting as an effective inhibitor of Mpro, a variety of protease necessary for proper viral replication, which prevents this process from taking place. Melatonin is also involved in the proper control of inflammatory and hyper-inflammatory processes, which is why it has a beneficial effect on acute lung injury and sepsis caused by the virus. Also, it has been observed that it reduces the side effects of antivirals such as ritonavir or lopinavir on the kidney and oxidative stress, reducing edema when associated with methylprednisolone. Therefore, these authors recommend melatonin as adjunctive therapy in the treatment of SARS-CoV-2 infection.

A study of 185 molecules was carried out for the treatment of COVID-19 in a somewhat more sophisticated contribution [39], among which stood out, for their better complementarity with matrix metallopeptidase 9 (MMP9), chloroquine and melatonin. MMP9 is related to neutrophil-mediated immune-inflammation, which acts as a membrane receptor for SARS-CoV-2, and, therefore, its ability to block this protein justifies the recommendation of melatonin for the treatment of COVID-19.

Other authors, although they insist on its key role in the suppression of COVID-19 infections due to its antioxidant properties, also point out other properties such as the ability to inhibit apoptosis [40], block lung inflammation, reduce alveolar edema, improve anxiety, prevent fibrosis, and stimulate immunity. These same authors [40] argue that the SARS-CoV-2 infection suppresses the production of mitochondrial melatonin, which induces a metabolic change that stimulates cytosolic glycolysis, which results in a reduction in the contribution of acetyl CoA, which acts as a precursor for endogenous melatonin synthesis. The decrease in the availability of melatonin consequently contributes to the production of the described “cytokine storm”, typical of this disease. Therefore, they recommend the administration of melatonin as an effective treatment against SARS-CoV-2 infection.

In this same line of argument, other authors [60] have postulated with their work that (1) melatonin is an excellent alternative in the management of viral infections due to its anti-inflammatory and antioxidant power and, therefore, it can alleviate the symptoms of SAR-CoV-2 infection. (2) They have observed that survival time increases by recovering the immune system and collaborating in the eradication of the virus. (3) They even state that supplementation with melatonin reduces the risk of COVID-19 incidence by attenuating respiratory distress and lung lesions, blocking apoptic mechanisms (pyroptosis), reducing oxidative stress, reducing cytokine storm, and improving distress. (4) With all of this, they come to affirm that melatonin has indirect antiviral actions in addition to reducing the risk of suffering from the COVID-19 disease.

Using computerized artificial intelligence techniques [61] focused on biological systems to screen available molecules and determine which are the most suitable for a specific treatment, in this work, it is stated that, in the case of COVID-19, those indicated are the pirfenidone and melatonin to reduce virus infection. Both drugs are safe and can be combined with other active ingredients as they do not present interactions, stating that they should be considered as highly recommended drugs in stage II patients at risk of severe pulmonary complications.

Currently, there is no definitive treatment against COVID-19, which makes its development an urgent need due to its serious repercussions. Consequently, Acuña-Castroviejo D et al. [45] propose melatonin as a molecule of great interest in the treatment of COVID-19 for the following reasons: (1) antioxidant function, which reduces the effects of free radicals, restores mitochondrial metabolism, and prevents lung damage; (2) anti-inflammatory function, caused by SARS-CoV-2 infection; (3) inhibitory of p65, preventing the transcriptional capacity and the inflammatory response. In the EducraCT clinical trial: 2008-006782-83, the authors demonstrate that the intravenous administration of 60 mg/day of melatonin improves the condition of patients and reduces their mortality and their hospital stay by up to 40%. To determine the dose to be administered, these authors designed a protocol that was approved by the Spanish Agency for Medicines and Medical Devices in a single-center, double-blind, randomized and placebo-controlled phase II trial, the objective being to address the efficacy and safety of intravenous administration of melatonin in patients admitted to the ICU.

In Zeng et al. [62], an integrative network-based deep learning methodology to identify reusable drugs for COVID-19 (termed CoV-KGE) is presented. This study demonstrates a powerful deep learning methodology to prioritize existing drugs for further investigation, which has the potential to accelerate therapeutic development for COVID-19. Melatonin plays a key role in regulating the human circadian rhythm that alters the translation of thousands of genes, including melatonin-mediated anti-inflammatory and immune effects against COVID-19. Among the antiviral activities that melatonin has, by suppressing multiple inflammatory pathways (e.g., IL6 and IL-1β), these effects are directly relevant given the well-described pulmonary pathophysiological characteristics of patients with severe COVID-19. This paper also highlights that the mechanism of action of melatonin may help explain the epidemiological observation that children, who have naturally elevated melatonin levels, are relatively resistant to COVID-19 disease manifestations, whereas older individuals, whose melatonin levels decline with age, are a very high-risk population. In addition, exogenous melatonin administration may be especially beneficial for older patients, given the age-related reduction in endogenous melatonin levels and the vulnerability of older individuals to SARS-CoV-2 lethality. This opens the door to new technologies such as deep learning both for the choice of new drugs and for a prior-refined bibliographic analysis on which to lay the foundations. We propose the design and implementation of a deep learning application focused on analyzing published studies on the performance of melatonin and its administration combined with other drugs as ongoing work.

Along with these considerations, we cannot forget other aspects related to SARS-CoV-2 infection, such as neurological manifestations and consequences [63,64,65,66] and, in this sense, the bibliography has already described different neuronal protection mechanisms and actions by melatonin, both in children and adults [67,68,69,70,71,72].

## 5. Conclusions

Once the contributions selected for this review were analyzed, we believe that the following considerations are relevant:

(1) Melatonin is a simple molecule, with well-documented pathophysiological functions, such as: the anti-inflammatory, antioxidant, and immunomodulatory action, as well as its inhibitory capacity of the Mpro protease and the MMP9 protein, which would make it a therapeutic alternative to consider against various infectious diseases.
(2) It has been known for years and it has been demonstrated once again with the administration of vaccines that the administration of melatonin can enhance the immune response, with a response in the rate of specific antibodies much higher than when the vaccine components are administered without melatonin.
(3) Before the onset of this pandemic, it had been shown in various viral infections that it could inhibit and/or mitigate the pathogenic action of these microbial agents in experimental animals.
(4) When melatonin is used in the laboratory (animal experimentation) and in the human clinic, a very wide safety margin has been demonstrated, well above most of the drugs used in ICUs against SARS-CoV-2 infection.
(5) We believe that there is a sufficient level of scientific evidence to authorize its use as a preventive drug against COVID-19 infection, due to its proven physiological actions, although it must be said that the exact dose to achieve this preventive effect has still not been determined.
(6) In infected patients with progressive disease, the scientific evidence is clear, and its administration is recommended for several reasons: (a) because it has been able to significantly reduce the consequences of the disease; (b) because there are no studies that say otherwise; (c) because its security profile is very broad.
(7) Although several administration guidelines have already been published in patients infected with SARS-CoV-2, it would be advisable to launch new clinical trials to define the best administration protocol, especially regarding dose and times when it should be administered, to respect its circadian rhythmicity.

## Figures and Tables

**Table 1:** Summary of Databases and Papers Reviewed.

Database	Articles Found	Articles Rejected for Content	Nº of Duplicate Items	Nº of Items Used
PUB MED	83	53	9	21
SCOPUS	18	9	4	5
DIALNET	42	30	5	7
SCIELO	27	19	2	6
GOOGLE SCHOLAR	27	23	3	1
Total	197	134	23	40

**Table 2:** Structured summary of each work analyzed.

Author/Journal	Objective	Type of Study	Q	Results	Conclusions
Brusco L et al. Melatonin Res. [42]	To analyze the therapeutic potential of melatonin to counteract the consequences of COVID-19 infection.	Review of clinical trials	A	The efficacy of melatonin at a dose of 9 mg/day is suggested to reduce the ICU stay of patients with pneumonia associated with COVID-19.	The importance of maintaining normal sleep and circadian rhythm in patients is confirmedInfected by COVID-19 in ICU.
Cardinali D, et al. Melatonin Res. [43]	Check for antigen reduction in individuals who experience partial or total loss of sleep prior to vaccination.	Longitudinal and prospective analytical observational.	A	The use of exogenous melatonin increases the potency of the immune response induced by the vaccine by increasing peripheral blood CD4 + T cells and B cells expressing Ig G	The administration of melatonin between 2 days before vaccination and 4 weeks after it may be an ideal complement to improve the effectiveness of vaccination against SARS-CoV-2.
Reiter RJ et al. Front Med (Lausanne). [48]	Identify safe and currently available molecules that can be used to slow or treat COVID-19 disease.	Longitudinal and prospective analytical observational.	A	Development of a therapeutic algorithm for the use of melatonin in patients with COVID-19	Melatonin should be considered for prophylactic use or treatment alone, or in combination with other drugs, and they propose a therapeutic algorithm for use in patients. In addition to its easy availability, it can be easily synthesized in large quantities, it is cheap, it has a very high safety profile, and it can be self-administered.
Zúñiga-Blanco BL, et al. Med Int Méx. [49]	Develop new guidelines to treat symptomatic patients and reduce viral load in asymptomatic carrier patients	Individual experimental study with sick subjects: clinical trial	M	Until effective antiviral treatment is available, early indication of prophylaxis with hydroxychloroquine is recommended.	In addition to cardiovascular support treatment, it is suggested to assess the indication of melatonin and tocilizumab to reduce clinical symptoms and the deterioration of patients with severe COVID-19
Ben-Nathan D, et al. Arch Virol. [50]	To study the effect of the pineal neurohormone melatonin (MLT) on protection against viral encephalitis	Experimental with mice	M	Injections of MLT to mice (10 PFU) reduced mortality in mice from 100% to 44%. By increasing the dose (100PFU) mortality decreased by 20%.	MLT’s Efficacy to Protect Against Lethal Viral Infections Warrants Further Investigation of Its Mechanisms of Action
Bahrampour Juybari K, et al. Virus Res [44]	To analyze the current evidence on the treatment of melatonin in viral infections	Longitudinal analytical observational	L	Melatonin promotes both humoral and cell-mediated immunity. Motivates synthesis of macrophage and granulocyte progenitor cells, NK cells and T helper cells specifically CD4 + cells	It is suggested that the use of melatonin in an outbreak of COVID-19 is beneficial, in the absence of a specific and effective treatment.
Sehirli AO, et al. Mol. Biol. Rep. [37]	To evaluate the importance of the CD147 protein and the possible protective effect of melatonin mediated by this protein.	Longitudinal analytical observational	M	CD147 is a glycoprotein responsible for the formation of the cytokine storm in the lungs through mediation of viral invasion. Melatonin reduces heart damage by blocking the activity of CD 147.	Melatonin is a safe drug that can prevent severe symptoms, reduce the severity of symptoms, and reduce the adverse effects of other antiviral drugs in COVID-19 patients.
Feitosa EL, et al. Int J Med Sci. [38]	To rationally identify new inhibitors of the main protease (Mpro) of SARS-CoV-2 using silico-tools, which show additional pharmacological properties against COVID-19	Molecular docking studies of binding sites and interaction energies of 74 Mpro-ligand complexes	A	59 impact compounds are identified, with melatonin standing out for its immunomodulatory and anti-inflammatory activity. The results do not confirm the antiviral activity of melatonin, but are the basis for clinical trials	The use of melatonin may have a response potential in early stages due to its possible effects on ACE-2 and Mpro, as well as in severe stages due to its action against hyperinflammation
Hazra S, et al. Life Sci. [39]	Explore the host protein (s) targeted by potent reused in COVID-19	Clinical trial with patient blood microarray data	A	Chloroquine and melatonin showed functions associated with neutrophil-mediated immunoinflammation	The present study reveals that between chloroquine and melatonin, melatonin appears to be a more promising repurposed drug against MMP9 for better immunocompromise in COVID-19.
Martín Giménez VM, et al. Nanomedicine (Lond). [40]	Carry out a new proposal based on the use of nanoformulated melatonin targeting mitochondria as a possible treatment for COVID-19	Descriptive basic research	L	Nanoformulation of melatonin offers advantages over conventional pharmaceutical preparations due to the kinetics of drug release, greater protection against early oxidation, and improves cellular absorption and bioavailability.	Based on the available technology, the administration of melatonin in nanoparticles should be analyzed as a new therapeutic alternative for the treatment of COVID-19 and/or other viral infections.
Öztürk G, et al. Turk J Med Sci. [51]	To analyze the benefits of melatonin as a treatment for COVID-19 in the elderly	Comparative descriptive study	L	Melatonin plays a fundamental role in the prevention of oxidative stress and mitochondrial dysfunction caused by free radical reactions initiated by mitochondria in the aging process	With age, melatonin levels decrease, so supplementation in the elderly could be beneficial in the treatment of COVID-19. More studies are required along this line.
Artigas L, et al. PLoS One. [52]	Combining Pirfenidone and Melatonin Medications to Identify Appropriate COVID-19 Treatment	Clinical trial in symptomatic patients of COVID-19 with different severity	A	Pirfenidone and melatonin are safe drugs that can be combined with the current standard of care treatments for COVID-19	The combination of pirfenidone and melatonin with standard COVID-19 treatment is considered of interest in patients at risk of developing serious pulmonary complications
Acuña-CastroViejo D, et al. J Pineal Res [45]	Determine the doses to be used and the efficacy of melatonin in SARS-CoV-2 infection	Phase II, double-blind, randomized, placebo-contrasted trial	M	Intravenous administration of 60 mg/d of a proprietary formulation of melatonin improved septic patients, reduced their mortality to zero and their hospital stay by 40%	Melatonin may be useful in the treatment of COVID-19 for the following reasons: (1) Its antioxidant action to reduce the effects of free radicals, restore mitochondrial metabolism, and prevent lung damage. (2) Anti-inflammatory function, caused by SARS-CoV-2 infection. (3) Inhibitor of p65, preventing the transcriptional capacity and the inflammatory response.
